# Application of Nanoparticles: Diagnosis, Therapeutics, and Delivery of Insulin/Anti-Diabetic Drugs to Enhance the Therapeutic Efficacy of Diabetes Mellitus

**DOI:** 10.3390/life12122078

**Published:** 2022-12-11

**Authors:** Tilahun Ayane Debele, Yoonjee Park

**Affiliations:** Department of Chemical & Environmental Engineering, College of Engineering and Applied Science (CEAS), University of Cincinnati, Cincinnati, OH 45221, USA

**Keywords:** diabetic mellitus, insulin, anti-diabetic agent, nanoparticles, drug delivery

## Abstract

Diabetes mellitus (DM) is a chronic metabolic disorder of carbohydrates, lipids, and proteins due to a deficiency of insulin secretion or failure to respond to insulin secreted from pancreatic cells, which leads to high blood glucose levels. DM is one of the top four noncommunicable diseases and causes of death worldwide. Even though great achievements were made in the management and treatment of DM, there are still certain limitations, mainly related to the early diagnosis, and lack of appropriate delivery of insulin and other anti-diabetic agents. Nanotechnology is an emerging field in the area of nanomedicine and NP based anti-diabetic agent delivery is reported to enhance efficacy by increasing bioavailability and target site accumulation. Moreover, theranostic NPs can be used as diagnostic tools for the early detection and prevention of diseases owing to their unique biological, physiochemical, and magnetic properties. NPs have been synthesized from a variety of organic and inorganic materials including polysaccharides, dendrimers, proteins, lipids, DNA, carbon nanotubes, quantum dots, and mesoporous materials within the nanoscale size. This review focuses on the role of NPs, derived from organic and inorganic materials, in the diagnosis and treatment of DM.

## 1. Introduction

### 1.1. Background

Diabetes mellitus (DM) is a group of chronic metabolic disorders resulting in high blood glucose (hyperglycemia) levels due to defects in insulin production or secretion, an increase in the production of glucagon, or resistance to insulin action which primarily impaired carbohydrate, lipids, and protein metabolism [1]. DM is one of the four top non-communicable diseases (NCDs), (i.e., cardiovascular diseases, cancers, chronic respiratory diseases, and diabetes) and has a great impact on public health and socio-economic development throughout the world [2,3]. The prevalence of DM is rapidly increasing throughout the world as a result of population aging, urbanization, environmental pollution, inappropriate diet, smoking, and low physical activity [4]. According to the World Health Organization (WHO), both the number of cases and the prevalence of diabetes have been progressively increasing from 108 million to 422 million in 1980 and 2014, respectively [5]. In addition, according to the International Diabetes Federation (IDF) reports, ~451 million adults (age 18–99 years) and ~463 million people were living with diabetes worldwide in 2017 and 2019, respectively, and this number is expected to increase to ~700 million by 2045 [6,7,8]. As a result of the high prevalence, the world faces a substantial economic burden in the form of higher medical costs (such as expenditure for insulin, testing strips, and treatment), less productivity, and premature mortality. The absolute global economic burden of DM was $1.3 trillion in 2015 and will be an estimated $2.1 trillion by 2030. In the United States only, the economic burden associated with diagnosed diabetes expenses was $327 billion in direct medical costs and $90 billion in lost productivity in 2017 [9]. In 2019, the expense of diagnosed diabetes was $760 billion which was supposed to increase because of the high DM prevalence [10]. 

### 1.2. Types of Diabetes Mellitus (DM)

Insulin, a peptide hormone secreted by the β cells of the pancreatic islets of Langerhans, controls peripheral glucose uptake (such as skeletal muscle and adipose tissue) via several families of glucose transporter proteins (e.g., GLUT4) and regulates glucose production within the liver (such as glycogenolysis (break down of storage glycogen), gluconeogenesis (synthesis of glucose from non-carbohydrate precursors)) [11]. Primarily, insulin-stimulated GLUT4 mediates exogenous glucose uptake by skeletal muscle where a large amount of glucose is stored in the form of glycogen and undergoes glycogenolysis when required to produce energy (ATP) [12]. However, in the absence of insulin or peripheral insulin resistance, GLUT4 remains intracellular within storage compartments (i.e., in muscle and fat cells) and alters glucose transport from the extracellular milieu into the cell which leads to hyperglycemia.

According to several kinds of literature most DM cases fall into three main categories: (i) Type 1 diabetes (insulin-dependent DM, due to β-cell destruction, usually leading to absolute insulin deficiency); (ii) Type 2 diabetes (non-insulin-dependent DM, due to insulin resistance in peripheral organs) and (iii) Gestational diabetes mellitus (GDM) (diabetes diagnosed in the 2nd or 3rd trimester of pregnancy). In addition to those major categories, there are also minor types of diabetes due to other causes, including, diseases of the exocrine pancreas (such as cystic fibrosis, pancreatitis, pancreatectomy), a genetic defect of β-cell function (such as neonatal diabetes and maturity-onset diabetes of the young) [13], a genetic defect of insulin action (such as leprechaunism, Rabson–Mendenhall syndrome, lipoatrophic diabetes) [14,15,16], endocrinopathies (such as acromegaly, Cushing’s syndrome, glucagonoma, pheochromocytoma, hyperthyroidism, somatostatinoma, aldosteronoma) [17] and drug or chemical-induced diabetes (such as glucocorticoids, statins, nicotinic acid, calcineurin inhibitors) [18]. 

Among those categories, about 90–95% of cases are related to type 2 DM which is the most prevalent throughout the world. The high prevalence of type 2 DM is primarily due to obesity, a sedentary lifestyle, and genetic predisposition [19]. As mentioned above, type 2 DM is caused by peripheral insulin resistance, where a normal or elevated insulin level produces an attenuated biological response and is followed by beta cell destruction which alters glucose homeostasis [20]. To achieve good metabolic control and delay type 2 DM, a combination of both lifestyle changes (such as exercise, and dietary intake) and pharmacological (oral and/or injectable drugs) treatment is required to improve insulin production, and function [21,22,23,24]. In addition, in the case of the complete absence of insulin, insulin replacement therapy is often required [25]. 

Type 1 DM also known as insulin-dependent diabetes or juvenile diabetes, accounts for 10% of all DM cases [26]. It is caused by the autoimmune destruction of pancreatic β-cells, resulting in an absolute deficiency of insulin production [27,28]. This condition is characterized by severe insulinopenia which leads to hyperglycemia. As a result, exogenous insulin therapy is required to maintain a basal level of insulin, prevent disease-related complications and preserve life. Insulin products have been categorized based on their activity profiles, as rapid-acting (such as insulin lispro, insulin aspart, and insulin glulisine), short-acting (such as regular soluble insulin), intermediate-acting (such as neutral protamine Hagedorn (NPH) insulin), long-acting (such as insulin glargine, insulin detemir, and insulin degludec), pre-mixed insulin and inhaled insulin (such as Afrezza) [29,30,31]. However, multiple injections of insulin per day are painful and inconvenient, resulting in poor patient compliance. As a result, most patients still have trouble maintaining ideal blood glucose levels through insulin replacement therapy [32]. 

The third type of diabetes category is GDM, which is most frequently diagnosed during the 2nd or 3rd trimester of pregnancy [33]. GDM is defined as any degree of glucose intolerance with onset or first recognition during pregnancy that was not present or recognized before pregnancy [34]. It is diagnosed when the pancreatic function in women is not enough to control the diabetogenic environment that pregnancy confers [35]. It is a common complication during pregnancy (~7% of all pregnancies are complicated by GDM) and the prevalence may range from 1 to 14% of all pregnancies. Research studies have shown GDM has many risk factors, including being overweight, obesity, oral contraceptive use, micronutrient deficiencies, or advanced maternal age, which are directly or indirectly correlated with impaired β-cell function and/or insulin resistance [36,37]. 

### 1.3. Diagnosis and Treatment of DM 

Diagnosis is the first crucial step for DM management. Conventional methods of diabetes diagnosis rely on analyzing fasting plasma glucose (FPG) levels, the oral glucose tolerance test (OGTT), and hemoglobin A1c (HbA1c) levels [38,39]. However, the conventional methods are considered painful by some patients due to the injection during blood withdrawal, while the measured value varies due to several factors such as age, time of testing, and physiological condition. Moreover, this method is unsuitable for continuous monitoring of DM patients due to the tedious process, long time to diagnosis, large amount of venous blood withdrawal, and whole blood processing [40]. Above all, the manifestation of DM symptoms such as hyperglycemia are clinically observed after disease onset, which prohibits early intervention. Recently, to address these weaknesses different types of nanotechnologies, such as nanobiosensors, and theranostic nanoparticles have been developed to potentially enable earlier and non-invasive detection of diabetes. 

Controlling blood glucose levels is extremely important for the prevention of DM-related complications, and to minimize premature morbidity and mortality associated with the disease. Chronic complications of DM may lead to the dysfunction of various major organs, such as eyes, blood vessels, heart, kidneys, and nerves which can increase mortality from infections, cardiovascular disease, stroke, kidney failure, leg amputation, foot ulcers, chronic liver disease, vision loss, and nerve damage. Mainly, long-term complications of DM are associated with high blood glucose levels (hyperglycemia) which lead to glycation and impairment of different types of proteins. In general, a primary goal of management in all types of DM is the maintenance of blood glucose levels within the healthy normoglycemic range of 72 to 99 mg/dL (4.0 to 5.4 mmol/L) during fasting, and up to 140 mg/dL (7.8 mmol/L) 2 h after eating. 

Currently, insulin therapy is crucial in the management of DM, mainly for type 1 and advanced type 2 DM, by regulating blood glucose level homeostasis and preventing associated chronic complications, morbidity, and mortality. However, multiple injections per day are required, because of the short half-life of insulin which is associated with pain, trauma, and distress to the patients [41,42]. In addition, subcutaneous administration of insulin has certain disadvantages including peripheral hyperinsulinemia, hypoglycemia, lipohyperatrophy, excessive weight gain, insulin neuropathy, and insulin presbyopia [43,44,45,46]. Thus, focusing on the alternative route of administration (oral or pulmonary) or minimizing the injection doses are required to overcome the poor patient compliance and drawbacks associated with this conventional method. However, biochemical (such as luminal pH, and GIT enzymatic degradation) or physical barriers (such as mucosa layer, intestinal epithelium, and tight junctions) are the major hindrances in insulin delivery (i.e., orally, or pulmonary) [47,48,49]. For example, intestinal epithelium permits (i.e., paracellular and transcellular passive diffusion) only lipophilic drugs with less than 700 Da in molecular weight, which limits insulin delivery due to its high molecular weight of 5800 Da. In addition, the tight junction is selectively permeable to small hydrophilic molecules (nutrients, ions, and certain drugs) and solutes of molecular radius < 1.5 nm (~3.5 kDa molecular weight) [50]. Hence solute with a molecular radius of more than 1.5 nm (>3.5 kDa molecular weight) is usually excluded from traversing this barrier [49]. 

Many efforts have been made to design better formulations for oral insulin delivery using different techniques, including: co-administration of protease inhibitors (such as Na-glycocholate, bacitracin, and camostat mesylate); the addition of absorption/permeation enhancers (such as bile salts, ethylene diamine tetraacetic acid, surfactants, fatty acids, and zonula occludens toxin (ZOT)); slight modifications of insulin (such as PEG conjugation or non-covalent interaction with organic/lipophilic carriers); conjugation of ligands (such as lectins, transferrin, immunoglobulins, folate, vitamin B12, epidermal growth factor) to target specific receptors at sites of interest; or conjugation of cell-penetrating peptides [51,52]. Although all these mentioned strategies showed promising results and improved the pharmacokinetic and pharmacodynamic characteristics of insulin, there are still certain limitations reported for absorption enhancers, such as long-term cytotoxicity and for protease inhibitors, protein malabsorption due to disturbed protein digestion.

**Figure 1:**
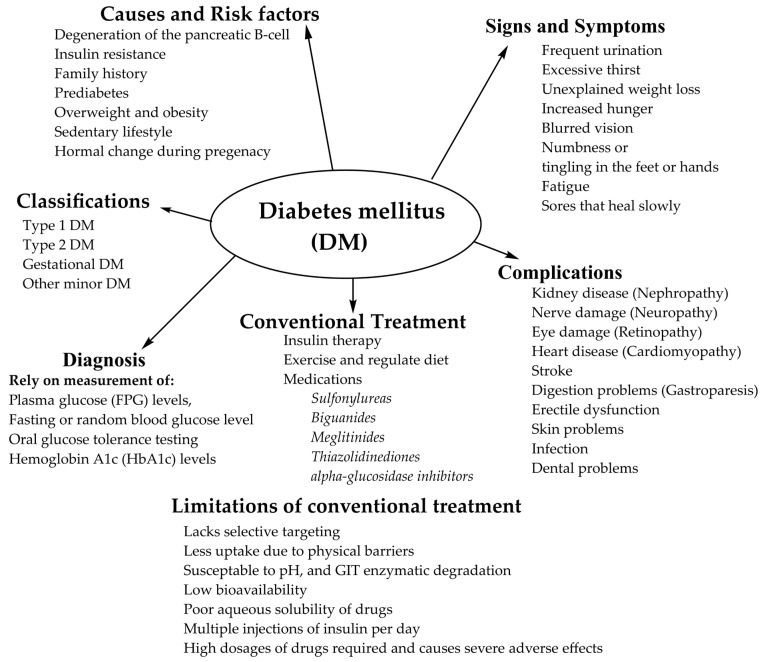
The etiology, pathophysiology, and conventional treatment methods of DM.

In addition to insulin, there are a lot of hypoglycemic therapeutic agents used to treat DM, mainly type 2 DM. Currently, five distinct classes of hypoglycemic agents are available for patients whose diet, and exercise do not offer satisfactory glucose control, which includes sulfonylureas, biguanides, meglitinides, thiazolidinediones, and alpha-glucosidase inhibitors. According to the Biopharmaceutical Classification System (BCS), most hypoglycemic agents belong to class II with low solubility and high permeability. Due to the poor aqueous solubility of hypoglycemic agents, their bioavailability and therapeutic efficacy are highly altered. As a result, high dosages of drugs, either single or combination agents, are required to maintain normal physiological blood glucose levels, which may cause severe adverse effects. Hence, to meet the typical physiological goals, mainly between meals and during the night, a faster drug release followed by a prolonged drug release profile over an extended period is required to regulate homeostasis of plasma glucose levels. Several researchers have reported a novel delivery system, which tends to overcome drug delivery challenges and improve bioavailability, which in turn enhances therapeutic efficacy with a lower therapeutic dose and better patient compliance. Although great achievements have been obtained, there are still certain limitations in the treatment and management of type 2 DM. Hence, a holistic strategy is needed, which focuses on the prevention and intervention of a novel therapeutic approach in drug delivery to enhance therapeutic efficacy and reduce DM-related chronic complications. The etiology, pathophysiology, and conventional treatment methods of DM are summarized in Figure 1.

**Figure 2:**
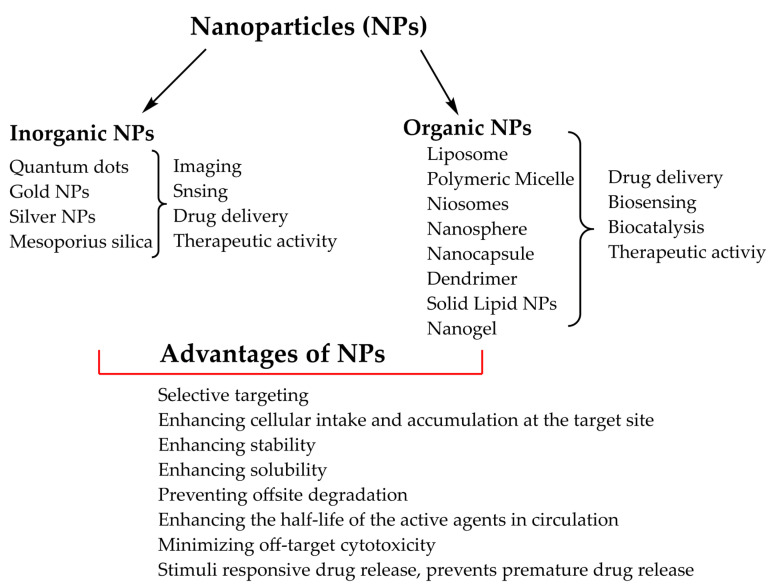
Classification, biomedical applications, and advantages of nanoparticles (NPs).

In the last two decades, nanotechnology has played a great role in several fields of medicine in diagnostic and therapeutic functions. Nanoparticles (NPs) have distinct physical, chemical, biological, and unique structural characteristics which makes them attractive for biomedical applications including drug delivery, medical imaging, sensing, diagnosis, and therapy [53]. Designing and synthesizing novel NPs are the primary pillars in nanomedicine to improve the targeting and accumulation of therapeutic/diagnostic agents at the site of interest or by directly interacting with the disease-causing pathogens as active therapeutics [54]. NPs have been synthesized from a variety of organic and inorganic materials (Figure 2) including polysaccharides, dendrimers, proteins, lipids, DNA, carbon nanotubes, quantum dots, and mesoporous materials within the nanoscale size [55]. These nanocarriers have been designed in different forms, including polymeric micelles, conjugates and complexes of dendrimers, hyperbranched polymers, prodrugs, inorganic NPs, polyplexes, lipopolyplex, liposomes, polymersomes, nanogels, and vesicles, and have been used to deliver pharmaceutical agents to a site of interest either by chemically conjugating, or physically encapsulating therapeutic agents [56]. 

In comparison with conventional therapeutic methods, nanocarrier-mediated drug delivery has more advantages in terms of selective targeting, enhancing cellular intake and accumulation, enhancing stability, preventing offsite degradation, and enhancing the half-life of the active agents in circulation, which in turn enhances the therapeutic efficacy of pharmaceutical agents only at the site of interest by minimizing off-target normal cytotoxicity [57,58]. In addition, several stimuli-sensitive nanocarriers have been synthesized which were important for delivering and releasing cargo at the site of interest based on the type of signals, either internal or external, within their constituent units [59]. The main principle of stimuli-sensitive nanocarriers is based on the fact that an endogenous (such as pH, redox, enzyme) or exogenous (such as temperature, photo, or ultrasound) stimulus can change the structural composition/conformation of the nanocarriers (such as carrier degradation, shell cleavage, charge reversal, and linker cleavage), thus promoting the release of the pharmaceutical agents at the site of interest precisely, in a temporal or spatial pattern [60,61,62]. 

In this review paper, the application of NPs in DM management, from diagnosis and disease monitoring to therapeutics will be discussed. Furthermore, the role of NPs in the delivery of insulin and other anti-diabetic drugs will be assessed. 

## 2. Application of Nanoparticles in the Diagnosis of DM

NPs can be used as diagnostic tools for the early detection and prevention of diseases owing to their unique biological, physical, optical, chemical, and magnetic properties [63]. For example, NPs can be used as an imaging probe in diseased tissues or organs, due to their nanoscale size or by using specific binding ligands on NP’s surfaces [64]. In addition, due to their longer circulation time and being more targetable in the body by changing their size, surface charge, and other properties, NPs are widely explored as contrasting agents for different types of biomedical imaging modalities, including magnetic resonance imaging (MRI) which can be used in the early diagnosis of DM [65,66]. Moreover, NPs have a large surface-to-volume ratio, which can offer a better matrix for the immobilization of enzymes and improve enzyme–substrate interactions by circumventing free enzyme aggregation, which in turn enhances enzymatic activity [67,68]. Several researchers have assessed different materials that can be used as matrices for enzyme immobilization, including magnetite NPs, gold NPs (AuNPs), and carbon nanotubes [69,70]. 

Overall, the synthesis of highly sensitive biosensors (such as nanosensors), as well as NPs that improve glucose sensor function, will ultimately improve the lives of patients living with DM. A biosensor is an analytical device and powerful diagnostic tool to detect the presence or concentration of a biological analyte [71]. It consists of three main parts: (i) components that recognize biological elements (such as enzymes, receptors, antibodies, nucleic acids, aptamer, microorganisms, and lectins) and produce signals (ii) signal transducer (such as electrochemical, optical, thermometric, piezoelectric, and magnetic) that converts the biorecognition event into a measurable signal, and (iii) a reader device that converts the signal into a readable form [72,73]. 

At present, glucose oxidase (GO) is the standard enzyme in glucose biosensors and it is widely used to measure glucose levels in biological samples based on the enzyme-catalyzed, oxidation mechanism [74]. In glucose biosensors, immobilized GO catalyzes the oxidation of β-D-glucose to gluconic acid in the presence of flavin adenine dinucleotide (FAD) as co-factors that are reduced to FADH2. The reduced FADH_2_ is regenerated by reacting with molecular oxygen, leading to the formation of hydrogen peroxides (H_2_O_2_) which are oxidized at a catalytic—classically platinum (Pt)—anode. Finally, the electrode recognizes the number of electron transfers, and this electron flow is proportional to the number of glucose molecules present in the sample, such as blood. Although glucose biosensors based on GO are widely used in clinical diagnosis due to their low cost, availability, good sensitivity, relatively good selectivity to glucose, and fast and real-time detection, it still has certain limitations including the poor electron transfer efficiency between the active center of GO and electrode materials [75]. Recently, several researchers have synthesized nanomaterials with excellent conductivity and biocompatibility (such as graphene, AuNPs, and conducting polymers) as electron transport media to improve the performance of enzyme-based biosensors [76,77]. In addition, the incorporation of NPs into the sensors provides a variety of advantages, including increased surface area, more efficient electron transfer from enzyme to electrode due to the excellent conductivity and small bandgap, enhanced stability, and the ability to include additional catalytic steps [78,79,80]. The most common application of nanotechnology for sensors in diabetes is the use of NPs to assist standard enzymatic electrochemical detection of glucose or direct detection of glucose oxidation at an electrode; i.e., nonenzymatic glucose sensors [81]. Integrating NP-based composites with enzymes provides a potent strategy to enhance biosensor performance, due to the unique physicochemical properties of NPs. Of these, recently, quantum dots (QDs) have been used as a preferable material in enzyme-based biological analyses and applications because of their good optical characteristics, high catalytic effects, high photostability, resistance to photobleaching, and high efficiency of electron-transfer. However, the QDs themselves do not interact with glucose, because it lacks inherent recognition ability and must be coupled to a recognition element for successful implementation. As a result, different researchers have tried to couple cadmium telluride (CdTe) QDs with glucose oxidase (GO). For example, Cao et al., have synthesized CdTe QDs-GO complex that can be used as a nanosensor for simultaneous assay of GO enzymatic activity (with a low Michaelis–Menton constant, 0.45 mM L^−1^) and glucose sensing with a detection limit of 0.10 μM [82]. Wang et al., also developed GO immobilized graphene–CdS (G–CdS) nanocomposite [83]. The author observed better electron transfer properties due to the synergistic effect of a graphene sheet and CdS nanocrystals. The synthesized glucose biosensor showed satisfactory analytical performance with a detection limit of 0.7 mM. Devasenathipathy et al., also synthesized GO immobilized AuNPs with graphene and multiwalled carbon nanotubes as amperometric glucose biosensors [84]. They reported that the amount of electroactive GO and the electron transfer rate constant were 10.5 × 10^−10^ mol cm^−2^ and 3.36 s^−1^, respectively. Similarly, Liu et al., developed a glucose biosensor based on AuNPs, GO, and polynorepinephrine [85]. The author reported that the synthesized biosensor showed high glucose sensitivity (low Michaelis–Menten constant (6.8 mM)) with a detection limit of 1.34 μM at a signal/noise ratio of 3. 

In addition to sensing and measuring glucose concentration, early assessment of certain biomarkers is important because individuals at high risk of developing type 1 diabetes can be identified several years before the clinical onset of the ailment. Biomarkers are biomolecules that are found in blood, tissue, or other body fluids and used as indicators of biological and pathological processes, or physiological and pharmacological responses to drug treatment [86]. For example, Lee et al., reported an ultrasensitive protein nanoprobe system that specifically captures disease markers (autoantibodies of type 1 diabetes) with attomolar sensitivity [87]. The system relies on supramolecular protein NPs that bind glutamate decarboxylase (GAD_65_)-specific autoantibody, (i.e., the early marker of type 1 diabetes). NPs were formed by intermolecular self-assembly between the chimera protein molecules and stable conformation per autoantibody binding, thereby allowing substantial enhancement of sensitivity, 4–9 orders of magnitude more sensitive than conventional immunoassays. This ultrasensitive protein nanoprobe successfully detected natural autoantibodies in the sera from type 1 diabetic patients. 

## 3. Application of Nanoparticles in Insulin Delivery

One of the ways to overcome insulin-related limitations, mentioned above, is formulating insulin using biocompatible and biodegradable nanocarriers which can resist gastric pH and protect insulin from enzymatic degradation and enhance intestinal residence. Cellular uptake of NPs is dependent on intrinsic (such as surface charge, shape, particle size, and mucoadhesive properties) and extrinsic (such as route administration) factors which determine the bioavailability and efficacy of nanoformulated drugs, including insulin. In addition to NPs nature, cellular uptake is also reliant on the physical characteristics of pharmaceutical agents, such as hydrophobicity, molecular weight, pH stability, and ionization constants. Thus, an understanding of biomolecules and these distinct mechanisms is important in designing delivery systems for oral protein drugs. As a result, many researchers are focused on optimizing nanocarrier design to protect macromolecules from GIT, pH and enzymes and also to prolong intestinal permeation which could improve the pharmacodynamics and pharmacokinetics characteristics of insulin [88]. Hence, different studies have been carried out using polymer-based (i.e., natural, or synthetic polymer) NPs for oral insulin delivery, and promising results were observed. 

**Figure 3:**
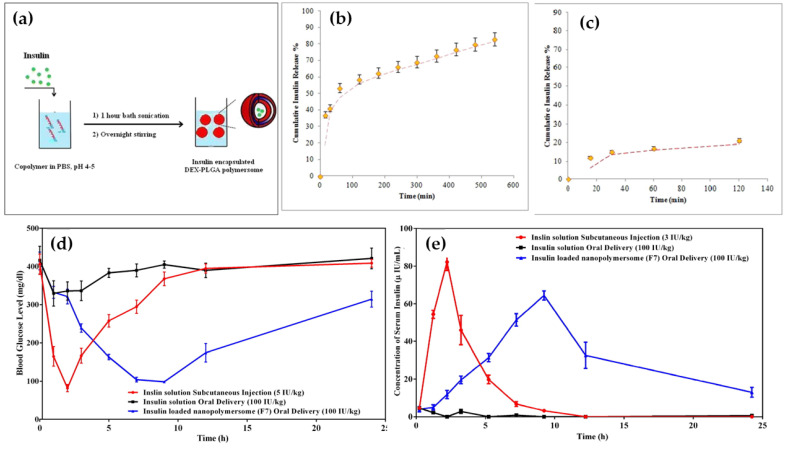
(**a**) Scheme of insulin encapsulation in polymersomes, (**b**) Insulin release profile at pH 7.4 from DEX5000-PLGA13000 polymersomes, (**c**) Insulin release profile at pH 1.2 from DEX5000-PLGA13000 polymersomes, (**d**) blood glucose level and (**e**) blood insulin concentration of diabetic rats after subcutaneous insulin solution injection at a dose of 5 IU/kg/b.w., orally administrated insulin solution at a dose of 100 IU/kg/b.w and orally administrated F7 formulation at a dose of 100 IU/kg/b.w. Reproduced with permission from Elsevier [89].

Alibolandi et al., synthesized insulin-encapsulated polymersomes based on amphiphilic copolymers of dextran-poly(lactic-co-glycolic acid) (DEX-PLGA) as shown in Figure 3 [89]. The synthesized polymersomes show high encapsulation efficiency for insulin (>90%). The in vitro insulin release study revealed significant insulin release in simulated intestinal conditions (pH 7.4), whereas non-negligible release was observed in simulated gastric (pH 1.2) conditions. In addition, a circular dichroism result shows, the secondary and tertiary structures of the released insulin were identical to that of standard insulin. Moreover, significant hypoglycemic effects were observed in the in vivo diabetic rat model which revealed the efficacy of the DEX-PLGA-based polymersomes as oral insulin carriers. 

Similarly, Ji et al., synthesized insulin-loaded nanocomposites (IN-Z-CSA), using chitosan and zein-carboxymethylated short-chain amylose, to improve the oral bioavailability of insulin [90]. The IN-Z-CSA revealed high encapsulation efficiency (89.6 ± 0.9%), and a good loading capacity (6.8 ± 0.4%). The author reported the transepithelial permeability of the IN-Z-CSA was 12-fold higher than that of insulin. Moreover, orally administered IN-Z-CSA had a stronger hypoglycemic effect with a relative bioavailability of 15.19%. 

**Figure 4:**
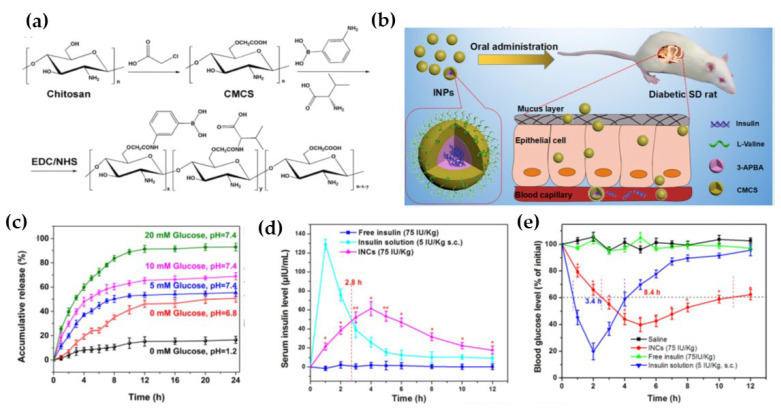
(**a**) Synthesis of chitosan-derivative, (**b**) Schematic illustration of chitosan-based multifunctional nanocarriers (**c**) The insulin release in vitro, from nanocarriers against SGF (pH 1.2) and SIF (pH 6.8) at different concentrations of glucose (at T = 37 °C) (**d**) Serum insulin level in diabetic rats following oral administration of free insulin, insulin solution and insulin-loaded nanocarriers (**e**) Blood glucose levels in diabetic rats following oral administration of saline, insulin-loaded nanocarriers, free insulin, and insulin solution. Reproduced with permission from Elsevier [98].

In addition, numerous researchers have synthesized NPs with specific ligands to enhance the cellular internalization efficiency via the receptor-mediated endocytosis pathway [91,92,93]. Some ligands such as lectins, RGD, biotin, and short peptides (such as CKSTHPLSC) have been reported to target different receptors in the intestinal epithelium cells (such as enterocytes, M cells, and goblet cells) [94,95,96]. Jin et al., prepared CSKSSDYQC (CSK) conjugated insulin-loaded NPs using trimethyl chitosan chloride (TMC) [97]. Compared with untargeted NPs, the CSK-targeted NPs showed better cellular uptake via clathrin and caveolae-mediated endocytosis on goblet cell-like HT29-MTX cells. After 2 h of oral administration, in diabetic rats, high plasma insulin concentration was observed for TMC-CSK INS NPs (62.87 ± 3.36 μIU/mL) in comparison to TMC INS NPs (39.57 ± 2.64 μIU/mL) in treated groups. In addition, the relative bioavailability of TMC-CSK INS NPs (5.66%) was 14.5-fold and 1.5-fold higher than that of insulin solution (0.39%) and TMC INS NPs (3.69%), respectively. Li et al., also synthesized L-valine targeted glucose-responsive chitosan-based multifunctional nanocarriers to deliver insulin as shown in Figure 4 [98]. The encapsulation efficiency (EE%) of insulin is about 67%. The in vitro insulin release study showed that the insulin release was much higher in simulated intestinal fluid (pH 6.8) than in simulated gastric fluids (pH 1.2). It may be because the carboxyl groups remain protonated at lower pH which restricts the swelling and subsequent release of loaded drugs. Furthermore, high insulin release was observed in the presence of a higher concentration of glucose (20 mM), than that of a low concentration of glucose (5 mM). Glucose molecules permeate into the inner nanocarriers, the negatively charged phenylboronic acid can combine with the 1, 2-diols of glucose and form stable and soluble phenyl borates, which shifts the equilibrium to the side for forming more combinations and results in the swelling of the nanocarriers and allows “triggered release” of insulin. Moreover, in vivo hypoglycemic studies showed, after oral administration of insulin-loaded nanocarriers to diabetic rats, an effective hypoglycemic effect was obtained compared with subcutaneous injection of insulin at the same concentration of insulin. 

**Figure 5:**
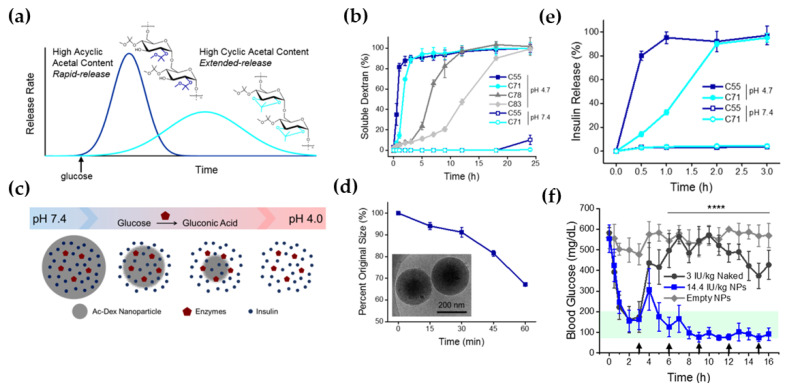
(**a**) Release kinetics from rapid-release (high acyclic acetal content) and extended-release (high cyclic acetal content) Ac-dex NPs, (**b**) Rate of degradation of Ac-dex NPs by percent cyclic modification, as determined by the concentration of soluble dextran in the supernatant. (**c**) Schematic of glucose-responsive insulin release from acid-degradable Ac-dex NPs, (**d**) Average relative diameter of C55NPs in acetate buffer showing a reduction in size over time. Inset: cryo-transmission electron micrograph of NPs at time 0. (**e**) Insulin release from C55NPs or C71NPs incubated in either acetate buffer (pH 4.7) or phosphate-buffered saline (PBS, pH 7.4) at 37 °C, (**f**) Blood glucose levels of streptozotocin-induced type 1 diabetic mice following administration of empty NPs, 3 IU/kg naked insulin, or 14.4 IU/kg insulin in Ac-dex NPs. Arrows represent intraperitoneal glucose tolerance tests (GTTs, 1.5 g/kg) every 3 h. Statistical significance is indicated by **** *p* < 0.0001. Reproduced with permission from the American Chemical Society [105].

Moreover, stimuli-responsive NP formulations have shown significant potential in solving the problems related to premature or off-target drug release due to their unique response to specific stimuli and release of their payloads only at the site of interest [99]. An insulin delivery system that self-regulates blood glucose levels would have great importance in preventing hypoglycemic events and improving glycemic control [100]. As a result, several researchers have tried to synthesize stimuli-sensitive (such as glucose-responsive, and pH-responsive), nanocarriers as an alternative approach to delivering insulin [101,102]. Synthesized nanocarriers are expected to respond to the local changes in glucose, which in turn trigger and control insulin release rates. Glucose-responsive nanocarriers can be synthesized by the covalent attachment of a glucose-sensing moiety, such as a boronic acid or its derivative, or by incorporation of the enzyme glucose oxidase (GO) and catalase into pH-sensitive biomaterials [100,103,104]. GO catalyzes glucose oxidation into gluconolactone which is readily hydrolyzed to gluconic acid (reducing the pH levels of surrounding environments) non-enzymatically, which could further trigger pH-sensitive nanocarriers to release insulin as a glucose-responsive system. Catalase is important for regenerating oxygen by decomposing hydrogen peroxide, formed as a byproduct by the GO enzyme, which is important for the catalytic conversion of glucose by the GO enzyme. Similarly, Volpatti et al., developed glucose-responsive acetylated dextran NPs (Ac-dex NPs) that showed rapid glycemic control, and prolonged normoglycemia for 16 h with a single subcutaneous injection of a diabetic mouse model as shown in Figure 5 [105]. 

**Figure 6:**
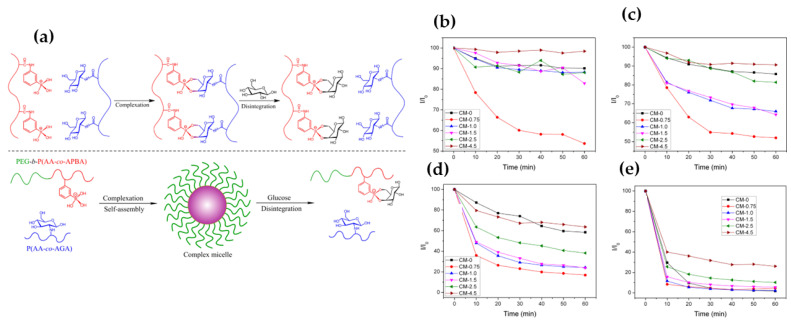
(**a**) Schematic illustration for the complexation and glucose-responsive disintegration of the PEG-b-P(AA-co-APBA)/P(AA-co-AGA) complex micelles. Glucose-responsiveness of the complex micelles in the presence of glucose with different concentrations, (**b**) 2, (**c**) 5, (**d**) 20, and (**e**) 50 g/L in aqueous solutions of PBS 7.4 in terms of normalized light scattering intensity as a function of time. Reproduced with permission from the American Chemical Society [109].

In summary, ideally, a glucose-responsive insulin delivery system could offer long-term glycemic control with reduced dosing frequency, respond rapidly and autonomously to changes in blood glucose levels, and prevent the incidence of hypoglycemic conditions. However, several kinds of literature revealed that GO-based glucose-responsive delivery significantly delays insulin release since the accumulation of gluconic acid must first overcome physiological buffering effects to reduce the pH of the surrounding microenvironment [106]. In addition, while encouraging results were observed, the enzymatic conversion of glucose in gluconic acid using GO is variable and slow due to the insufficient oxygen supply in vivo, which affects insulin release from the nanocarriers. As a result, alternative, non-enzymatic glucose-responsive, delivery systems are widely explored including covalent attachment of a glucose-sensing moiety such as boronic acid (BA) or its derivative phenylboronic acid (PBA), which reversibly react with diol compounds such as glucose, which leads to swelling of polymeric matrices and facilitates insulin release [107]. Several reports show that boronic acid-containing NPs are much more stable and suitable for long-term storage than GO-based drug carriers [108]. Ma et al., synthesized glucose-responsive micelles using a phenylboronic acid-based block copolymer PEG-*b*-P(AA-*co*-APBA) and a glycopolymer P(AA-*co*-AGA) [109]. The author reported that the complex micelles were stable under physiological conditions (PBS 7.4, 150 mmol) while they dissociated in the presence of glucose due to the replacement of glycosyl groups by free glucose as shown in Figure 6. However, there are certain limitations related to the use of boronic acid, including, lack of specificity for glucose, higher affinity for other diols such as fructose, and hindering glucose binding at physiological pH (7.3) because of its high pKa (~9.0) value. To overcome these limitations, PBA derivatives are developed that can function under physiological pH with improved glucose specificity [110,111,112]. 

## 4. Application of Nanoparticles in the Delivery of Other Hypoglycemic Agents 

**Figure 7:**
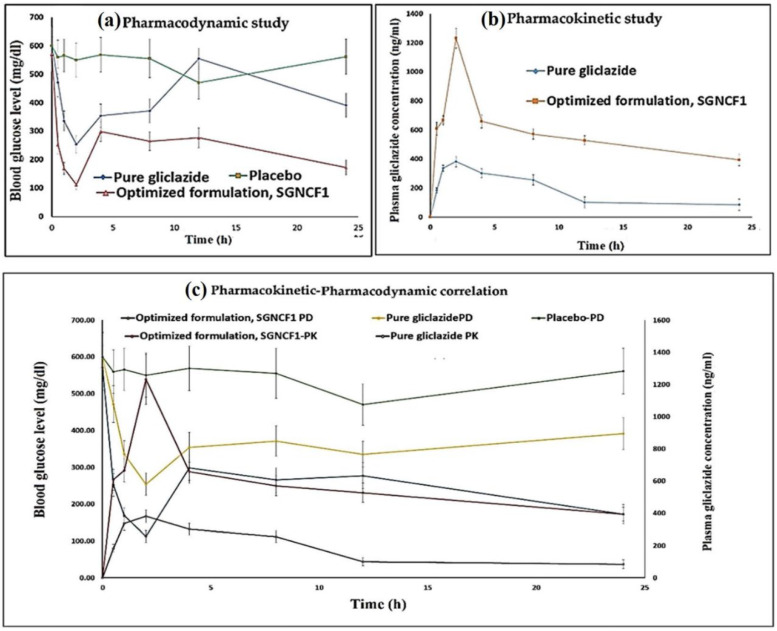
In vivo study of gliclazide loaded PLGA SGNCs formulation, (**a**) Pharmacodynamic responses of pure gliclazide, placebo and optimized formulation, SGNCF1 on type II diabetic rats, (**b**) Plasma mean gliclazide concentration–time curves after a single oral dosing of pure gliclazide and optimized formulation, SGNCF1 on type 2 diabetic rats, (**c**) Pharmacokinetic–pharmacodynamic correlation plot of gliclazide loaded PLGA SGNC formulation on type 2 diabetic rats. Reproduced with permission from Springer nature [115].

The bioavailability and therapeutic efficacy of hypoglycemic agents are highly altered, due to their poor aqueous solubility. Hence, a novel delivery system that improves bioavailability and therapeutic efficacy with low therapeutic doses is required [113,114]. One of the novel delivery systems is NP based formulations that exhibit remarkable improvements in solubility, drug loading capacity, cellular uptake, bioavailability, off-target drug release, off-target cytotoxicity, and therapeutic efficacy with a low dose of the hypoglycemic drug. Panda et al., have synthesized second-generation smarter PLGA-based nanocrystals (SGNCs) to encapsulate gliclazide, a BCS class II drug used in type 2 DM, using a combined method of emulsion diffusion, high-pressure homogenization, and solvent evaporation [115]. Compared to pure gliclazide, the solubility, dissolution rate, and bioavailability of gliclazide SGNCs were significantly improved. In vitro drug release showed an initial burst of gliclazide release, followed by prolonged release with better bioavailability that facilitates efficient delivery with better therapeutic effect as shown in Figure 7. Rani et al., also synthesized glycyrrhizin- and metformin-loaded NPs via the ionotropic gelation method, using chitosan and gum arabic as polymers [116]. The author reported that comparable to free metformin, lower dosage of nanoformulated metformin—approximately 1/4th—showed an equivalent anti-diabetic effect. Similarly, Nazief et al., synthesized Solid Lipid NPs (SLNs) loaded gliclazide via an ultra-sonication technique using glyceryl behenate (compritol^®^888 ATO) as a lipid matrix and poloxamer 188 (PLX) as a stabilizer [117]. Unlike the immediate release of free gliclazide, the biphasic *in-vitro* release with an initial burst followed by a prolonged release was observed for SLN loaded gliclazide. Pharmacokinetics results showed, five-fold increased oral bioavailability of SLNs loaded with gliclazide compared to free gliclazide powder. In addition, pharmacodynamics results showed, SLN loaded gliclazide has a better anti-diabetic action than the free gliclazide powder. Moreover, upon repetitive oral administration, SLN loaded gliclazide proved to be safe at doses equivalent to 15–20 times the human dose.

Moreover, Baig et al., have synthesized vildagliptin-loaded triangular DNA nanospheres coated with eudragit (Eud–DNA–VG) via the electrostatic attraction method [118]. The formulated nanospheres have good drug entrapment efficiency of up to 95 ± 2% and extended drug release of up to 15 ± 2 h. The ex-vivo studies indicated that Eud–DNA–VG nanospheres effectively bypass the acidic pH of the stomach and enhance glycemic control in Db/Db mouse without any risk of pancreatitis or pancreatic cancer.

In general, as discussed in the Section 3 and Section 4, different form of NPs plays great role in the improvement of therapeutic efficacy of encapsulated insulin or other hypoglycemic drugs by enhancing their bioavailability at the target site. As summarized in Table 1, several nanocarriers, with different composition, can be used to encapsulate and deliver several forms of drugs to improve their efficacy both in the in vitro and in vivo studies. 

## 5. Application of Nanoparticles as Therapeutic/Anti-Diabetic Agents 

Macro and micro minerals play a key role in the proper functioning of proteins, many enzymes, and transcriptional factors. For instance, Zn, Mg, and Mn are cofactors for several enzymes [128,129,130]. Zn and Cr are involved in the synthesis and secretion of insulin from the pancreatic beta-cells and increase the insulin receptor activity on target tissues, respectively [131,132,133]. The insulin molecule forms polymers with zinc in β-cell granules and zinc plays a major role in the secretion of insulin from pancreatic β cells [134,135]. The antidiabetic and insulin-like effect of zinc has been reported in several in vitro and in vivo studies [136]. Zn enhances the phosphorylation of the insulin receptor β-subunit and successively stimulates the phosphatidyl inositol 3-kinase and protein kinase [137,138]. As a result, of the several kinds the literature showed, Zn oxide NPs (novel agent to deliver Zn) have great implication for DM treatment [139,140,141,142]. El-Gharbawy et al., have synthesized ZnONPs with a median size of ∼20 nm via the sol-gel method using zinc acetate and methanol as precursors to revive the function and structure of β-cells [143]. ZnONPs alone or together with vildagliptin (DPP-IV inhibitor) significantly decreased microRNA-103 and microRNA-143 expression (potential biomarkers for type 2 DM) compared to the control diabetic group, indicating its antidiabetic effects. In addition, ZnONPs improved many indices of diabetic dysfunction (such as glucose tolerance, weight loss, insulin levels, fructosamine levels, pancreatic SOD activity, and pancreas histology) and have a significant antidiabetic effect. The anti-diabetic properties of ZnONPs were briefly discussed by Kim San Tang in his review paper entitled “The current and future perspectives of zinc oxide NPs in the treatment of DM” [144].

In addition, several researchers reported that inorganic NPs have a promising potential in treating DM due to their anti-oxidative activity against reactive oxygen species (ROS) [145]. ROS (e.g., superoxide anion (O_2_^•−^), hydroxyl radical (OH^•^), hydrogen peroxide (H_2_O_2_), and hypochlorous acid (HOCl)) are derivatives of molecular oxygen or partially reduced metabolites of oxygen that own strong oxidizing capabilities [146]. At physiological concentrations, ROS play a great role in cell metabolism and as a signaling molecule that regulates cell growth and differentiation [147,148]. However, at high concentrations ROS oxidizes and damages cellular macromolecules (such as protein, lipid, and DNA) that ultimately affect their biological function, leading to cell apoptosis and necrosis [149]. Moreover, when a greater imbalance occurs in favor of the ROS, oxidative stress results, which is identified as the causal factor for cardiovascular disease, diabetes, rheumatoid arthritis, cancer, and neurodegenerative disorders [150,151]. It is, therefore, advantageous to employ exogenous antioxidants or agents which can control the level of ROS through different chemical reactions.

Currently, several NPs with catalytic activity toward ROS are designed and used to protect against oxidative stress-related toxicity. For example, Weaver et al., have synthesized enzyme-mimetic auto-catalytic, antioxidant, self-renewing cerium oxide nanoparticle (CONP)-composite hydrogel encapsulated β-cells [152]. Although cytotoxicity was observed after phagocytosis of low concentrations (~1 mM) of CONPs by β-cells, the authors reported that co-incubation of CONPs with β-cells has potent cytoprotection from superoxide exposure. In addition, after CONPs-composite hydrogel was embedded within alginate hydrogels, an excellent cytoprotection effect from the free radical attack was observed without cytotoxicity, to encapsulated β-cells at up to 10 mM concentration of CONPs. Similarly, BarathManiKanth et al., have synthesized gold nanoparticles (AuNPs) to control the hyperglycemic conditions in streptozotocin-induced diabetic mice [153]. The author reported that AuNPs tend to inhibit lipid peroxidation and ROS generation during hyperglycemia. In addition, the AuNPs exhibited an insistent control over the blood glucose level, lipids, and serum biochemical profiles in diabetic mice near the control mice provoking their effective role in controlling and increasing organ functions for better utilization of blood glucose. 

In addition, manganese-based NPs have an antioxidant effect and could aid in the survival and continued function of the embedded cells by reducing free radical load and improving oxygenation. MnO_2_ NPs display numerous useful properties including high reactivity with H_2_O_2_ and the capability to fully recover the oxygen, which makes them the best choice in glucose-responsive materials. Wu et al., used manganese oxide NPs to catalytically remove H_2_O_2_ produced through the oxidation of glucose in their self-regulating insulin release device [154,155]. Similarly, Tootoonchi et al., have synthesized and characterized four manganese oxide nanoparticles and evaluated their catalytic ability to remove ROS and produce oxygen, acting as in vitro enzyme mimetics [156]. The synthesized manganese NPs have minimal cytotoxicity to murine insulinoma cells and protect cells from H_2_O_2_ exposure. 

On the contrary, different studies showed that the minerals imbalance can be a cause of DM, primarily correlated with ROS generation which finally ends up in oxidative stress that will decrease the insulin gene promoter activity and mRNA expression in pancreatic islet cells [157,158]. Compared with the liver, β-cells have low antioxidant (i.e., 1% catalase, 2% glutathione peroxidase, and 29% superoxide dismutase (SOD) activities and as a result are more susceptible to oxidative stress [159,160,161]. Like free metals, their derivative NPs have multifaced effects on DM. The foremost common negative outcome associated with the therapeutic application of NPs is the excessive generation of ROS which is considered a key factor in metal NP-induced toxicity. This shows that metal oxide NPs have a dual effect on DM treatments, and hence their antioxidant or pro-oxidant properties must be assessed before being used to treat DM. 

In addition to metallic NPs, there are also a lot of research efforts to synthesize or extract natural compounds that have antidiabetic activities. Of these, phenolic compounds, such as catechin, epicatechin, epicatechin-3-gallate, epigallocatechin, epigallocatechin-3-gallate, and rosmarinic acid, have received great attention as inhibitors of α-glucosidase activities [162,163,164]. The inhibition of the function of the α-glucosidase enzyme could be one of the effective approaches to reduce blood glucose levels by delaying carbohydrate digestion. Several researchers reported that phenolic compounds such as a monomers and/or polyphenolics, can delay carbohydrate digestion and reduce blood glucose levels via inhibition of mucosal α-glucosidase activity [165]. Suner et al., reported that catechins and poly(catechin) NPs inhibit α-glucosidase enzyme activity [166]. They found that poly(catechin) NPs have better inhibitory effects on carbohydrate digestion, even at low concentrations, in comparison to the catechins monomer. Similarly, Sahiner et al., also synthesized poly(rosmarinic acid) NPs via an emulsion crosslinking method, that have potential to remain in the gut longer than the soluble rosmarinic acid monomer [167]. They reported that, poly(rosmarinic acid) NPs inhibit the α-glucosidase enzyme, 8.6-fold higher, at much lower concentration (0.12 mg/mL concentration almost inhibits 100%), than a similar concentration of rosmarinic acid monomer (inhibits only 60% of α-glucosidase activity). Hence, poly phenolic NPs have the capability of substituting the current α-glucosidase inhibitors, acarbose and miglitol, in the treatment of type 2 DM. 

## 6. Summary and Future Perspective

Insulin therapy and other hypoglycemic drugs are a vital tactic to control the blood glucose levels of diabetic patients and prevent associated chronic complications, morbidity, and mortality. For the last few decades, great efforts have been made to improve insulin and or hypoglycemic drug delivery to manage DM-related complications. Although certain improvements have been attained, still the currently available therapy is unsatisfactory. To improve the current conventional treatments, researchers are trying to synthesize novel NPs to formulate insulin and other anti-diabetic drugs. Recently, several insulin formulated nanocarriers have undergone clinical trials, even though many of them failed for clinical use due to high cytotoxicity, low level of oral bioavailability, and an elevated intraindividual difference in insulin absorption. This showed that various features such as particle size, surface charge, and internal chemical composition of NPs must be considered before designing and formulating insulin or other hypoglycemic drugs. In addition, the detailed interaction of nano-GIT, pharmacokinetics, and pharmacodynamics of nanoformulated anti-diabetic agents must be taken into consideration. Most probably soon, nanoformulated insulin or other antidiabetic drugs could replace the current DM treatment methods. 

## Figures and Tables

**Table 1:** Some selected nanocarriers based on insulin and other hypoglycemic drugs’ delivery to treat DM.

Type of Nanocarriers	Compositions	Encapsulated Drugs	Particle Size (nm)	In Vitro/In Vivo Studies	Key Findings	References
Liposome	Distearoylphosphatidylethanolamine-polyglyceline, dipalmitoylphosphatidylcholin, dipalmitoylaphosphatidylglycerol, cholesterol or stearyl amine.	Glucagon-like peptide-1 (GLP-1)	130 to 210	Rats	Improved hypoglycemic effects, increased insulin secretion and enhanced serum GLP-1 levels	[119]
Polymeric Micelle	Phenylboronic acid (PBA), poly(ethylene glycol)-b-poly(aspartic acid-co-aspartamidophenyl boronic acid) PEG-b-P(Asp-co-AspPBA) and a PAsp-based glycopolymer poly(aspartic acid-co-aspartglucosamine) P(Asp-co-AGA) w	Insulin	167 to 255	293T or NCI-H460 cells	Glucose responsiveness and on–off release of insulin was obtained under physiological pH 7.4 with 2 g/L glucose (hyperglycemia) as a trigger.	[120]
Niosomes	span-20 and cholesterol	pioglitazone (PTZ)	145.3 to 545	Rats	Increased blood glucose lowering potential compared to PTZ	[121]
Niosomes	Span 40, Chol with or without DOTAP or DCP	Metformin	223.5 to 384.6	Rats	Enhanced hypoglycemic effect compared to metformin	[122]
polymeric NPs	Poly (lactide-co-glycolide) [PLGA] and Poly (methyl methacrylate) (PMMA)	Metformin	<300	Rats	<5% hemolytic effect and less organ cytotoxicity	[123]
polymeric NPs	Poly (lactide-co-glycolide) [PLGA]-gambogic acid-GA), PLGA-GA2	Curcumin and Insulin	∼270	Rats	Significant decrease in the hind paw pad area of the diabetic group, preserved axonal neurites	[124]
Solid lipid NPs (SLN)	Sodium cholate, soya bean lecithin, tripalmitin, steric acid, Pluronic F68,	Insulin and GLFEAIEGFIENGWEGMIDGWYG (HA2) peptide	150–170	Caco-2 cells	Effectively escaped insulin from acidic endosome, significant hypoglycemic response (*p* < 0.05)	[125]
Solid lipid NPs (SLN)	Compritol, oleic acid, Tween 80 and Span 20	Myricitrin	76.1	Mice	Reducing oxidative stress and increasing antioxidant enzyme levels	[126]
Nanocomposites	Chitosan and Porous silicon	GLP1 and dipeptidylpeptidase-4 (DPP4)	172 to 223	Rats	32% reduction in blood glucose levels and ~ 6.0-fold enhancement in pancreatic insulin content was observed compared to the GLP-1 + DPP4 inhibitor solution	[127]

## Data Availability

Not applicable.
